# Purification and Characterization of a Novel Pentadecapeptide from Protein Hydrolysates of *Cyclina sinensis* and Its Immunomodulatory Effects on RAW264.7 Cells

**DOI:** 10.3390/md17010030

**Published:** 2019-01-06

**Authors:** Wei Li, Shengwang Ye, Zhuangwei Zhang, Jiachao Tang, Huoxi Jin, Fangfang Huang, Zuisu Yang, Yunping Tang, Yan Chen, Guofang Ding, Fangmiao Yu

**Affiliations:** Zhejiang Provincial Engineering Technology Research Center of Marine Biomedical Products, School of Food and Pharmacy, Zhejiang Ocean University, Zhoushan 316022, China; liweizjou@163.com (W.L.); yesw1003@163.com (S.Y.); all_zzw@163.com (Z.Z.); T1528358025@163.com (J.T.); jinhuoxi@163.com (H.J.); gracegang@126.com (F.H.); abc1967@126.com (Z.Y.); cyancy@zjou.edu.cn (Y.C.); dinggf2007@163.com (G.D.)

**Keywords:** *Cyclina sinensis*, protein hydrolysates, immunomodulatory peptides, RAW264.7 cell

## Abstract

In the present study, peptide fractions of *Cyclina sinensis* hydrolysates, with molecular weight (MW) < 3 kDa and highest relative proliferation rate of murine macrophage cell line RAW 264.7, were purified by a series of chromatographic purification methods, to obtain peptide fractions with immunomodulatory activity. The amino acid sequence of the peptide was identified to be Arg-Val-Ala-Pro-Glu-Glu-His-Pro-Val-Glu-Gly-Arg-Tyr-Leu-Val (RVAPEEHPVEGRYLV) with MW of 1750.81 Da, and the novel pentadecapeptide (named SCSP) was synthesized for subsequent immunomodulatory activity experiments. Results showed the SCSP enhanced macrophage phagocytosis, increased productions of nitric oxide (NO), tumor necrosis factor-α (TNF-α), interleukin-6 (IL-6), and interleukin-1β (IL-1β), and up-regulated the protein level of inducible nitric oxide synthase (iNOS), nuclear factor κB (NF-κB), and NOD-like receptor protein 3 (NLRP3) in RAW 264.7 cells. Furthermore, the expression of inhibitor of nuclear factor κB-α (IκB-α) was down-regulated. These findings suggest that SCSP might stimulate macrophage activities by activating the NF-κB signaling pathway and can be used as a potential immunomodulatory agent in functional food or medicine.

## 1. Introduction

The immune system plays a crucial role in maintaining human health by identifying and killing pathogens, aging cells, and tumor cells. Its functions can be affected by many factors, including social pressure, negative emotion, unhealthy lifestyle, and presence of pathogens [1]. Macrophages are vital immune cells that participate in both non-specific (innate immunity) and specific defense (cellular immunity) in vertebrates. Activated macrophages have been reported to directly kill and eliminate pathogens, necrotic cell fragments, and foreign substances via phagocytosis, and indirectly participate in immune regulation through the secretion and release of pro-inflammatory cytokines (TNF-α, IL-6, and IL-1β) and inflammatory molecules (reactive oxygen species (ROS), NO) [2] Macrophage activation is considered a promising strategy for enhancing host immune function. Currently, several drugs such as levamisole, imiquimod, pidotimod, tilorone, and cyclosporine are being clinically used for regulating human immune function. However, most immunomodulating drugs synthesized microbially or chemically, exhibit toxicity and side effects, which limits their usage in the prevention and treatment of chronic diseases [3]. Dietary therapy is considered an effective strategy for regulating immune function, as naturally derived immunomodulatory peptides do not show side effects.

Bioactive peptides (consisting of 2–20 amino acids) are protein fragments released from protein molecules via enzymatic hydrolysis and a series of refining processes, which harbor multiple bioactivities, including immunomodulatory [4], anti-inflammatory [5], antihypertensive [6,7,8], anticancer [9], antioxidant [10,11], and antifatigue [12] activities. Recently, several immunomodulatory peptides have been purified and identified from protein hydrolysates of natural origin [13]. Three immunomodulating peptides, Asn-Gly-Met-Thr-Tyr, Asn-Gly-Leu-Ala-Pro, and Trp-Thr, isolated from enzymatic hydrolysates of Alaska Pollock frame enhanced lymphocyte proliferation [14]. Wu, et al. [15] isolated a novel hexapeptide (Glu-Cys-Phe-Ser-Thr-Ala) from wheat germ globulin, which showed significant immunomodulatory effects on RAW 264.7 macrophages. However, few immunomodulatory peptides have been purified from proteins of crustaceans and mollusks.

*Cyclina sinensis*, a marine bivalve mollusk, widely cultured along the mud-sandy coasts of China, has been used for treatment of asthma, dental ulcers, and inflammation in Chinese traditional medicine [16,17]. Furthermore, *Cyclina sinensis* is rich in protein and polysaccharides [17], which may contribute to its biological properties, such as anti-cancer, immunomodulatory, antioxidant, and hepatoprotective activities [18,19,20,21]. Zhang, et al. [22] observed that *Cyclina sinensis* flesh extracts stimulated immune response and enhanced body resistance by increasing the activity of acid-naphthyl acetate esterase (ANAE) in macrophages and lymphocytes in aged mice. Yu, et al. [23] isolated a novel anti-proliferative pentapeptide (Ile-Leu-Tyr-Met-Pro) from protein hydrolysates of *Cyclina sinensis*, which induced apoptosis of DU-145 prostate cancer cells. The *Cyclina sinensis* -derived angiotensin-I converting enzyme (ACE) inhibitory peptide WPMGF (Trp-Pro-Met-Gly-Phe, 636.75 Da) shows potent ACE inhibitory activity with relatively stable physiological activity under different temperatures, pH, and simulated gastrointestinal digestion [8].

Previously, we have shown that pepsin hydrolysates of *Cyclina sinensis* with MW < 3 kDa significantly promoted the proliferation of RAW 264.7 murine macrophages [21]. However, the amino acid sequence of the immunomodulatory peptide was not confirmed, nor was its immunomodulatory effect accurately evaluated. Therefore, in this study, the protein hydrolysates of *Cyclina sinensis* with MW < 3 kDa were purified using chromatographic methods, and the amino acid sequence of the immunomodulatory peptide was identified using Edman degradation and electrospray ionization (ESI) mass analyses. The immunomodulatory activity on RAW 264.7 murine macrophages was evaluated for determining its capacity for phagocytosis, secretion of NO, TNF-α, IL-1β, and IL-6 was assayed, and the protein levels of iNOS, IκB-α, NF-κB, and NLRP3 were determined using western blotting. Our results indicated that SCSP can be potentially used as an immunotherapeutic adjuvant in functional food or medicine.

## 2. Results and Discussion

### 2.1. Purification of Activity Peptide

**Figure 1:**
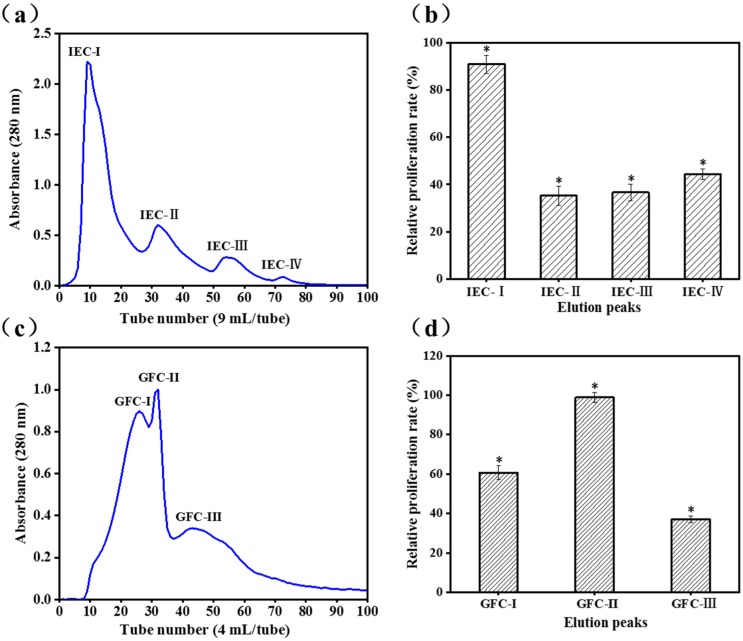
The peptide fraction of the *Cyclina sinensis* hydrolysate with MW < 3 kDa were purified using IEC and GFC; (**a**) Elution profiles of the fraction with MW < 3 kDa by anion exchange chromatography; (**b**) Effects of the elution fractions of IEC on the viability of RAW 264.7 cells; (**c**) Elution profiles of IEC-I by Gel filtration chromatography; (**d**) Effects of the elution fractions of GFC on the viability of RAW 264.7 cells; Data are presented as the mean ± SD (n = 3). (*) Results are significantly different from the control (*P* < 0.05).

DEAE Sepharose Fast Flow is a weak anion exchanger, and fractions with positive charges are eluted first when the hydrolysates are bonded to the exchangers [24]. Kong et al. [25] reported that higher content of positively charged peptides effectively stimulates immunomodulatory activity and is positive correlations. The ion-exchange chromatogram is shown in Figure 1a. The cell proliferative properties of the four fractions (IEC-I, IEC-II, IEC-III, and IEC-IV) obtained from the fraction with MW < 3 kDa based on its charge properties were determined at concentrations of 100 μg/mL. The relative proliferative rate of IEC-I (90.9 ± 3.8%) was twice of that IEC-II (35.2 ± 4.0%), IEC-III (36.6 ± 3.5%), and IEC-IV (44.4 ± 2.3%) towards RAW 264.7 cells (Figure 1b). Thus, peak IEC-I was selected for further purification using Gel-25 filtration chromatography.

Gel filtration (size exclusion) chromatography excludes high MW fractions from cross-linked agarose, which are first eluted and separated. The low MW fraction enters the interior and is subsequently separated [26]. IEC-I was fractioned into three subfractions of GFC-I, GFC-II, and GFC-III (Figure 1c) by the Sephadex G-25 gel filtration column and cell proliferative rates were determined at concentrations of 100 μg/mL. GFC-II possessed higher cell proliferative rate (98.9 ± 2.7%) toward RAW 264.7 cells than GFC-I (60.8 ± 0.3%) and GFC-III (37.1 ± 1.7%) (Figure 1d). A previous study also confirmed that immune cell proliferation correlated with the MW of peptides [25].

**Figure 2:**
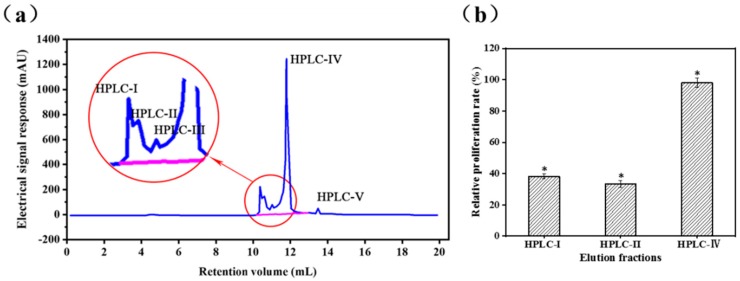
Purification of fractions GFC-II using RP-HPLC; (**a**) Elution profiles of GFC-II by Reverse phase high-performance liquid chromatography; (**b**) Effects of the elution fractions of RP-HPLC on the viability of RAW 264.7 cells; Data are presented as the mean ± SD (n = 3). (*) Results are significantly different from the control (*P* < 0.05).

The GFC-II fraction was further purified in an RP-HPLC system, the elution profile of which is shown in Figure 2a. Fractions HPLC-I, HPLC-II, and HPLC-IV were separately collected and lyophilized for measurement of cell proliferation rate. Owing to the low content of HPLC-III and HPLC-V, minor fractions from small peaks were not collected. Fraction HPLC-IV (98.16 ± 2.9%) not only possessed higher immunomodulatory activity, but also higher content than HPLC-I (38.2 ± 1.5%) and HPLC-II (33.4 ± 2.1%) (Figure 2b). Thus, HPLC-IV was collected for purity testing and determination of amino acid sequences

### 2.2. Analysis of Peptide Purity and Peptide Sequencing

**Figure 3:**
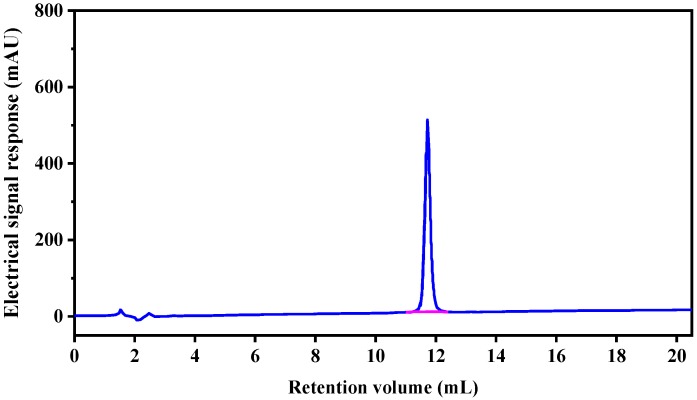
Purity analyze of HPLC-IV fraction by RP-HPLC.

**Figure 4:**
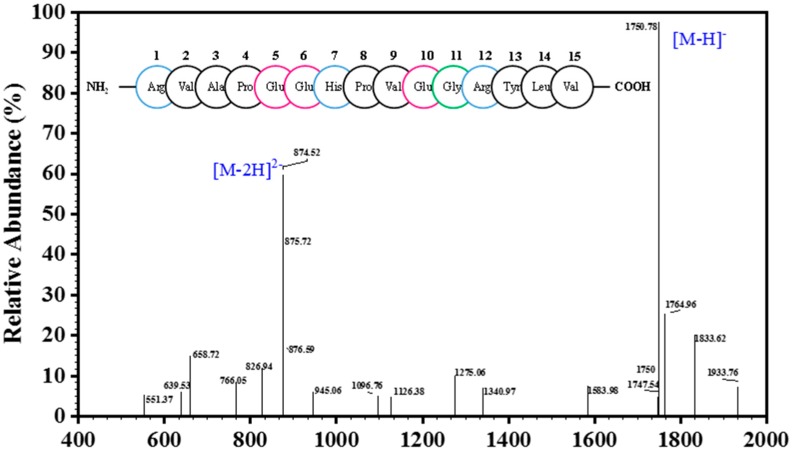
ESI-MS spectrum of the peptide CSP; The circles of different colors in the figure represent the amino acid sequence of CSP, wherein blue represents basic amino acid, black represents hydrophobic amino acid, red represents acidic amino acid, and hydrophilic amino acids are represented by green.

Only one peak was obtained in the elution profile of the HPLC-IV fraction during purity analysis (Figure 3), which was collected, lyophilized, and named CSP. The amino acid sequence of the purified peptide CSP was determined to be Arg-Val-Ala-Pro-Glu-Glu- His-Pro-Val-Glu-Gly-Arg-Tyr-Leu-Val (RVAPEEHPVEGRYLV) with a MW of 1750.78 Da ([M−H]−, Figure 4), which was consistent with a theoretical mass of 1750.96 Da. Hydrophobicity is an important physical property of proteins. Some studies have demonstrated that hydrophobic values can effectively contribute to the immunomodulatory activity of peptides [27,28]. This may be due to an increase in the interaction of the peptide and cell membrane, which improves immune regulation [29]. In addition, a terminal rich in basic or hydrophobic amino acids can also be considered as an indicator of immunomodulatory activity [15,29,30]. In this novel pentadecapeptide, the carboxy (-Arg-Tyr-Leu-Val) and amino termini (Arg-Val-Ala-Pro-) have basic and hydrophobic amino acids, respectively, and these characteristics may contribute to immunomodulatory activity.

### 2.3. Immunomodulatory Effects of SCSP

#### 2.3.1. Effect of SCSP on Macrophage Viability

**Figure 5:**
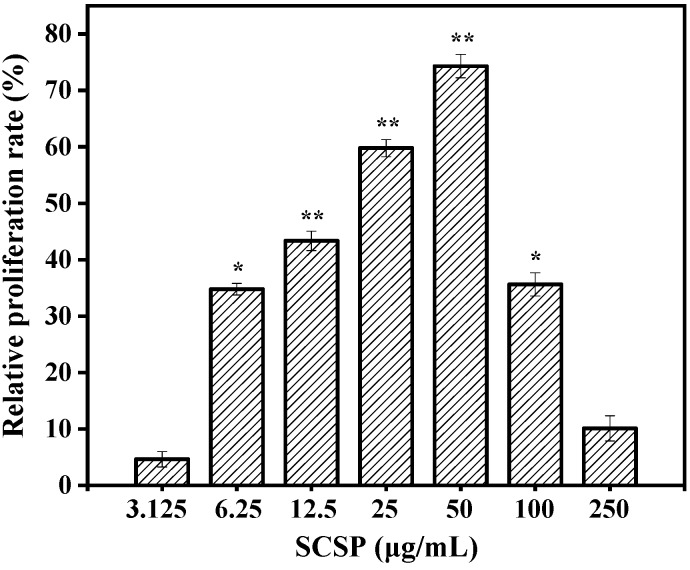
Effect of SCSP on macrophages viability. The significance of the subsequent data were presented as (*). Results are significantly different from the control (*P* < 0.05). (**) Results are significantly different from the control (*P* < 0.01).

Activated macrophages play critical roles in cell-mediated and humoral immunity. They first remove necrotic debris from tissues and cells at the site of injury via phagocytosis and then produce NO to directly kill the pathogen. Various cytokines and enzymes are secreted simultaneously, which participate in the inflammatory reaction and guide the process of body repair [31]. Therefore, macrophage viability can be used as an indicator of immunomodulatory effects and toxicity of an immune activator. The toxic effects on RAW 264.7 cells were examined using the MTT viability assay prior to investigating the immunomodulatory activity of SCSP. The results of the MTT assay (Figure 5) showed that SCSP treatment of promoted RAW 264.7 cell proliferation to a certain degree, indicating that the macrophages were activated. Compared to the control, 6.25–50 μg/mL SCSP significantly increased the viability of macrophages in a dose-dependent manner (*P* < 0.05). The relative proliferation activity of the cells showed a slight decrease when the concentration of SCSP was ≥ 100 μg/mL, indicating that high concentrations of SCSP showed slight cytotoxic effect on RAW 264.7 cells. The cytotoxicity exhibited by high concentrations of immunomodulators has also been reported [32,33]. Therefore, we treated RAW 264.7 cells with 12.5–50 μg/mL SCSP during subsequent experiments.

#### 2.3.2. Effect of SCSP on Phagocytosis

**Figure 6:**
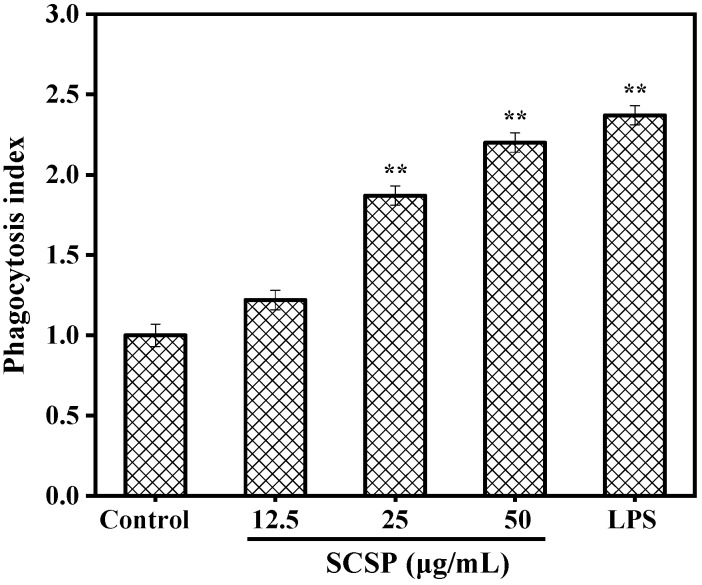
Effect of SCSP on phagocytic activity. The significance of the subsequent data were presented as (**). Results are significantly different from the control (*P* < 0.01).

Phagocytosis is important for specific and non-specific immunity, clearance of senescent or apoptotic cells and necrotic tissue fragments, and processes related to host defense and autoimmunity [34]. One of the most striking features of activated macrophages is the uptake of exogenous particles via phagocytosis [35]. Therefore, in the present study, we determined the effect of SCSP on the phagocytic activity of RAW 264.7 cells by constructing a model of neutral red dye internalization by macrophages. As shown in Figure 6, all phagocytosis indices of SCSP exceeded 1.0 and increased in a dose-dependent manner at various concentrations. The phagocytosis index reached 2.20 at the SCSP concentration of 50 μg/mL, which was slightly lower than that induced by 1 μg/mL LPS (2.32). In a previous study, Huang et al. [36] observed that oviductus ranae hydrolysate prepared with a neutral protease significantly regulated macrophage phagocytosis in a dose-dependent manner, and similar phagocytic activity was also observed in wheat germ globulin [15], Alaska pollock frame [37], and oyster (*Crassostrea gigas*) hydrolysates [3]. These data indicate that SCSP was able to enhance the phagocytic activity of macrophages in a dose-dependent manner.

#### 2.3.3. Effect of SCSP on NO Production and iNOS Expression

**Figure 7:**
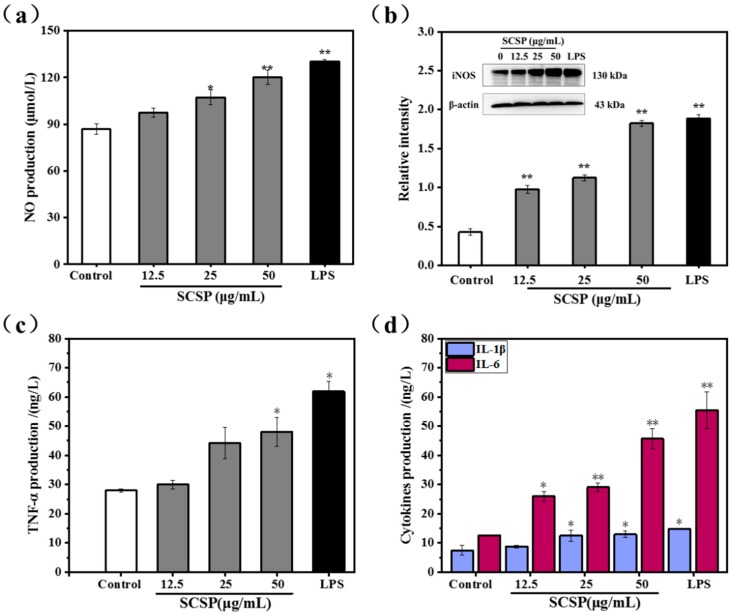
Assessment of release of nitric oxide and cytokines by macrophages with SCSP-treated; (**a**) Effect of SCSP on NO production; (**b**) Effect of SCSP on iNOS expression; (**c**) Effect of SCSP on TNF-α secretion; (**d**) Effect of SCSP on IL-1β and IL-6 secretion. The significance of the subsequent data were presented as (*). Results are significantly different from the control (*P* < 0.05). (**) Results are significantly different from the control (*P* < 0.01).

NO acts as a key messenger molecule for activated macrophages and plays an important role in immune regulation. It not only protects against exogenous pathogens and tumor cells, but also regulates cellular activities associated with the immune system [38]. Mammalian NO synthase (NOS) has three different isoforms: neuronal (nNOS), inducible (iNOS), and endothelial (eNOS). iNOS is easily induced and expressed in activated macrophages and is the most important enzyme in NO synthesis [39,40]. Hence, we assessed macrophage activation by measuring NO secretion in SCSP-treated macrophages. The protein level of iNOS was also analyzed using western bloting. As shown in Figure 7a, SCSP stimulated NO production in RAW 264.7 cells in a dose-dependent manner. At low concentrations (25 μg/mL), SCSP did not significantly affect NO production. However, NO production was significantly stimulated in RAW 264.7 cells at both medium (50 μg/mL, *P* < 0.05) and high doses (100 μg/mL, *P* < 0.01). In addition, the protein levels of iNOS increased remarkably after SCSP treatment (*P* < 0.05) (Figure 7b). These results demonstrated that SCSP up-regulated the protein levels of iNOS and NO secretion in RAW 264.7 cells in a dose-dependent manner, which is consistent with that previously demonstrated for oviductus ranae [36], yellow field pea seeds [41] and Coix glutelin protein hydrolysates (≤3 kDa) [42].

#### 2.3.4. Effect of SCSP on TNF-α, IL-1β and IL-6 Levels

Macrophage activation is one of the most important steps in anti-infection inflammatory responses and immune responses. Activated macrophages directly kill and eliminate pathogens and foreign substances via phagocytosis when stimulated by invasion or damaged by exogenous pathogens. Subsequently, iNOS is induced and NO is released, whereas pro-inflammatory cytokines and inflammatory molecules such as peptide growth factor (PGF), interleukin (IL), tumor necrosis factor (TNF), platelet-derived growth factor (PDG), and NO are secreted during immune response [43]. Considering the important role of cytokines in mediating and modulating immune and inflammatory responses, we assessed the immunomodulatory effects of SCSP by determining the secretion of TNF-α, IL-1β, and IL-6 by RAW 264.7 cells. The release of TNF-α was promoted in SCSP-treated RAW 264.7 cells in a dose-dependent manner, with significant increase in TNF-α level at 100 μg/mL SCSP (Figure 7c). Furthermore, IL-6 and IL-1β secretion was significantly enhanced by SCSP-treatment (Figure 7d). Previous studies on innate immune modulators have emphasized the effects of heteropolysaccharides isolated from Smilax *glabra Roxb* [44], polysaccharide ASKP-1 purified from *Artemisia sphaerocephala Krasch* seed [45], and the immunomodulatory protein PEP 1b from *Pleurotus eryngii* [46] on macrophage activation and secretion of IL-6, TNF-α, and IL-1β. These results indicated that SCSP displays significant immunomodulatory activities as it increases the secretion of NO, IL-6, IL-1β, and TNF-α in RAW 264.7 cells.

#### 2.3.5. Effect of SCSP on NF-κB and NLRP3

**Figure 8:**
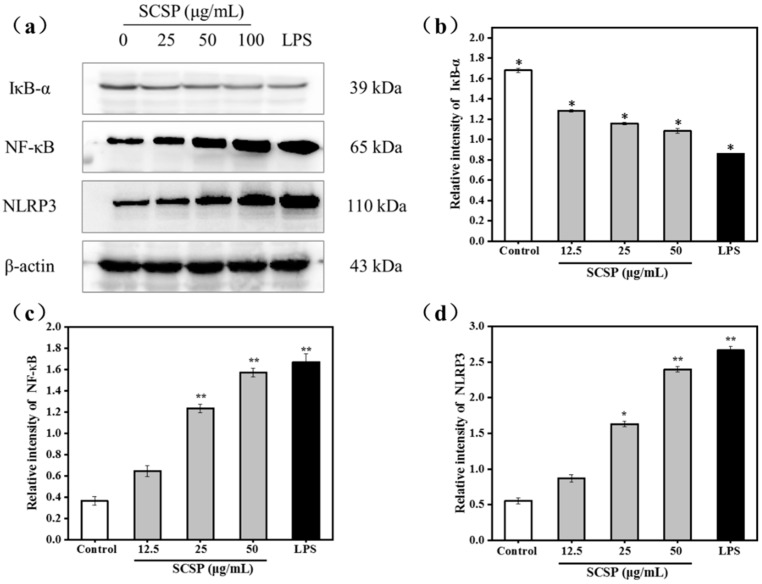
Effects of SCSP on the expression of IκB-α, NF-κB and NLRP3; (**a**) Western blot analysis of IκB-α, NF-κB and NLRP3 induced by SCSP in RAW264.7 cells; (**b**) Effects of SCSP on the expression of IκB-α in RAW264.7 cells; (**c**) Effects of SCSP on the expression of NF-κB in RAW264.7 cells; (**d**) Effects of SCSP on the expression of NLRP3 in RAW264.7 cells; The intensity of specific bands was detected by the Quantity One software (version 4.62) to quantify protein expression levels; The grayscale ratio of the specific bands and β-actin were presented as the mean ± SD (n = 3) by the histogram; (*) Results are significantly different from the control (*P* < 0.05). (**) Results are significantly different from the control (*P* < 0.01).

NF-κB is an indispensable transcription factor that regulates inflammatory gene expression and immune system function, and plays a vital role in inflammatory and immune responses [47]. IκB is an important member of the NF-κB signaling pathway and is responsible for regulating NF-κB activation and transcription. Among the IκB family proteins, IκBα mainly mediates basal inhibition of NF-κB activity [48]. The NF-κB dimer binds to the inhibitory protein IκBα, which sequesters the dimer in the cytoplasm in an inactive state in resting cells. However, when various stimulating factors (such as LPS, viral protein, oxygen free radicals, pro-inflammatory cytokines) stimulate cells, the cascade signal rapidly activates IκB kinase (IKK). Activated IKK phosphorylates Ser-32 and Ser-36 of IκB. Phosphorylated IκB-α is ubiquitinated by ubiquitin conjugation enzyme and is subsequently degraded by the proteasome, releasing NF-κB. Activated NF-κB enters the nucleus and regulates transcription of genes involved in immunity and inflammation (such as iNOS, TNF-α, IL-1β, and IL-6). As shown in Figure 8b, the NF-κB/β-actin ratio dramatically increased from 0.365 ± 0.04 (control group) to 1.669 ± 0.08 (*P* < 0.05) after treatment with 50 μg/mL SCSP, whereas that of IκB-α displayed an opposite trend in a dose-dependent manner (Figure 8a). This is because the cytoplasmic NF-κB translocated to the nucleus after degradation of IκBα post SCSP stimulation.

NLRP3 is the most widely studied intracellular receptor in the NOD-like receptor family [49]. Upon receipt of the induction signal, the apoptosis-associated speck-like protein (ASC), pro-caspase-1, and NLRP3 assemble into the NLRP3 inflammasome. The NLRP3 inflammasome activates caspase-1, which converts the pro-inflammatory cytokines pro-IL-1β and pro-IL-18 into IL-1β and IL-18, resulting in their secretion [49,50]. To investigate the effect of SCSP on NLRP3 inflammasome activation, we treated cells with LPS and 12.5, 25, and 50 μg/mL SCSP and determined the protein level of NLRP3 (*P* < 0.05). Our results illustrated that 50 μg/mL SCSP significantly promoted the expression of NLRP3, similar to that observed for LPS-treated RAW 264.7 cells (*P* < 0.05). SCSP induced the expression of NLRP3 in a dose-dependent manner. This is also evident from the increase in the levels of IL-1β in the supernatant obtained after SCSP treatment (Figure 8d). Therefore, we propose that SCSP elevated the production of NO, TNF-α, IL-1β, and IL-6 in activated macrophages via the NF-κB and NLRP3 inflammasome signaling pathways (Figure 8a).

## 3. Materials and Methods

### 3.1. Materials and Reagents

*Cyclina sinensis* was purchased from the Laoqi market in Zhoushan China, and validated by Prof. Shenglong Zhao of Zhejiang Ocean University (Zhoushan, Zhejiang, China). Pepsin was purchased from YTHX Biotechnology Co., Ltd. (Beijing, China). Acetonitrile and trifluoroacetic acid (high-performance liquid chromatography grade) were purchased from Shanghai Weston Trading Co., Ltd. (Shanghai, China).

Dulbecco’s modified Eagle medium (DMEM), fetal calf serum (FCS), and penicillin−streptomycin were purchased from Gibco BRL (Grand Island, NY, USA). Lipopolysaccharide (LPS), dimethyl sulfoxide (DMSO), and 3-(4, 5-Dimethylthiazol-2-thiazolyl)-2,5-diphenyl-2*H*-tetrazolium bromide (MTT) kits were purchased from Sigma-Aldrich Trading Co., Ltd. (St. Louis, MO, USA). Assay kits for IL-6, IL-1β, TNF-α, and NO were all obtained from Nanjing Jiancheng Bioengineering Institute (Nanjing, China). Neutral red and *N*-acetyl-l-cysteine (NAC) were purchased from Beyotime Institute of Biotechnology (Shanghai, China). Antibodies against β-actin (cat. no. 13E5), iNOS (cat. no. D6B6S), IκBα (cat. no. 44D4), NF-κB p65 (cat. no. D14E12), and NLRP3 (cat. no. D4D8T) were obtained from Cell Signaling Technology (Boston, MA, USA). All other reagents used were of analytical grade.

### 3.2. Purification of Immunomodulatory Peptide

We prepared the protein hydrolysates of *Cyclina sinensis* according to the method of Ye et al. [21], and the fraction with MW < 3 kDa was lyophilized using the alpha 1–4 LD plus freeze dryer (Marin Christ, Germany) for further purification. In detail, the pretreated *Cyclina sinensis* was treated with pepsin for 8 h at 42 °C (pH 2.0, material-liquid ratio 1:10 g/mL, enzyme dosage 1600 U/g), and then the pepsin was inactivated in boiling water for 15 min and centrifuged at 12,000× *g* for 15 min at 4 °C, and then Cogent μScale TFF ultrafiltration system (Merck Millipore, Burlington, MA, USA) with MW cut-off membranes of 3 kDa was used to separate the CSH fraction (Freeze-dried and stored at −80 °C until use). The target immunomodulatory peptide was purified from protein hydrolysates of *Cyclina sinensis* via ion-exchange, gel filtration, and reverse phase high performance liquid chromatography. The relative proliferation rate of RAW264.7 cells was used as index to purify the immunomodulatory peptide.

#### 3.2.1. Ion-Exchange Chromatography (IEC)

The more active fraction was dispensed into 0.05 M Tris-HCl buffer, pH 7.5 (2 mL, 200 mg/mL), which was added to a pre-equilibrated DEAE Sepharose Fast Flow column (7.1 × 25 cm, GE Healthcare Bio-Sciences AB, Uppsala, Sweden), and stepwise eluted using ultrapure water and NaCl solution of different concentrations (0.1, 0.5 and 1.0 M) at a flow rate of 6.0 mL/min. Fractions (9 mL/tube) were collected and detected at 280 nm, which were subsequently pooled and lyophilized for determining their cell proliferation activity toward RAW 264.7 cells at a concentration of 100 μg/mL.

#### 3.2.2. Gel Filtration Chromatography (GFC)

The fraction purified in the previous step (400 mg) was dissolved in 2 mL ultrapure water and purified using the Sephadex G-25 gel filtration column (8.0 × 48 cm, GE Healthcare Bio-Sciences AB, Uppsala, Sweden), which had been equilibrated previously with ultrapure water. Ultrapure water was used as an eluent at a flow rate of 1.0 mL/min; 4 mL of the eluate was collected per tube and detected at 280 nm. Three sub-fractions were collected and lyophilized for determining their cell proliferation activity toward RAW 264.7 cells at a concentration of 100 μg/mL.

#### 3.2.3. Reverse Phase High Performance Liquid Chromatography (RP-HPLC)

The fraction with highest cell proliferation activity was further purified using RP-HPLC (Agilent 1260 HPLC with auto fraction collector system) on a ZORBAX SB-C18 (5 μm, 9.4 × 250 mm, Agilent Technologies, Santa Clara, CA, USA) column with gradient elution system based on 100% ultrapure water as solvent A and 100% acetonitrile as solvent B. The gradient procedure was initiated with 100% solvent A after 3 min of holding, decreased to 30% solvent A within 5 min, and subsequently maintained at a flow rate of 2.0 mL/min for 10 min. The purification was repeated more than 30 times at the same gradient elution condition and the single peak fraction was collected. N-EVAP112 nitrogen evaporator (Organomation Associates, Berlin, MA, USA) was used to separate acetonitrile from the eluent. After that, the fraction was lyophilized and their cell proliferation activity against RAW 264.7 cells and their amino acid sequences were determined.

### 3.3. Determination of Amino Acid Sequence and Molecular Mass

The immunomodulatory peptide was subjected to N-terminal amino acid sequencing on a PPSQ-31A protein sequencer (Shimadzu Corporation, Kyoto, Japan). The mass spectrometer (ZQ2000, Waters, Milford, MA, USA) combined with an ESI source was used to measure the MW of the final purified peptide. Based on the sequence results determined in the previous step, Mimotopes Biotechnology Co., Ltd. (Wuxi, Jiangsu, China) provided us with peptides synthesized using the Fmoc-solid phase method for subsequent experiments.

### 3.4. Immunomodulatory Activity Analysis in RAW264.7 Cell Line

#### 3.4.1. Culture of RAW 264.7 Cells

Murine macrophage cell line RAW 264.7 was purchased from the Chinese Academy of Sciences Cell Bank (Shanghai, China). The cells were cultured in DMEM supplemented with 10% FCS containing 100 U/mL penicillin and 100 μg/mL streptomycin in an incubator with a humidified 5% CO_2_ atmosphere at 37 °C.

#### 3.4.2. Cell Viability Assay

The effects of SCSP on cell viability were determined using the MTT assay per the method described by Chen et al. [51]. Briefly, RAW 264.7 cells in the logarithmic growth phase were seeded in a 96-well plate (1 × 10^4^ cells/mL, 200 μL/well) and continuously cultured for 12 h in a 5% CO_2_ incubator (Forma 3111 CO_2_ incubator, Thermo Forma, USA) at 37 °C. Then, the medium was discarded, and the cells were treated with different concentrations of SCSP (250, 100, 50, 25, 12.5, 6.25, and 3.125 μg/mL). LPS (1 μg/mL, dissolved in DMEM) and DMEM were used as the positive and control, respectively. After culturing for 36 h, 200 μL MTT (5 mg/mL) was added to each well and incubated for another 4 h, the supernatant was removed, 150 μL of DMSO was added to each well, and incubated with mild agitation in the dark for 10 min at room temperature for complete dissolution of the produced MTT formazan crystals. The optical density (*OD*) of each well was measured at 490 nm using an automatic microplate reader (SpectraMax M2, Molecular Devices, San Jose, CA, USA).

The relative proliferation rate (%) was computed according to the following equation:

$$\mathrm{Relative}\;\mathrm{proliferation}\;\mathrm{rate}\;\left(\%\right)=\left[\left(OD_{treated}-OD_{control}\right)/OD_{control}\right]\times 100$$

where *OD_treated_* is the *OD* value of the cells treated with SCSP or LPS; *OD_control_* is the *OD* value of the cells cultured in DMEM without treatment.

#### 3.4.3. Phagocytosis of Neutral Red

We used the method described by Ren et al. [52] with slight modifications to determine the effects of SCSP on the phagocytosis of RAW 264.7 cells. After the cells in 96-well plates were cultured with different concentrations (100, 50, and 25 μg/mL) of SCSP and LPS (1 μg/mL) for 36 h, the cell culture medium was removed and 0.1% neutral red solution was added at 100 μL/well, followed by incubation for 3 h. The cells cultured in DMEM were used as blank group, which was untreated with SCSP or LPS. Excess neutral red in the culture solution was removed by washing twice with 0.01 M PBS (pH 7.4), 200 μL lysis solution (ethanol/acetic acid 1:1) was added per well, and the plates were shaken for 10 min at 25 °C. The *OD* of each well was measured at 540 nm using a microplate reader.

The phagocytosis rate was calculated using the following equation:

$$\mathrm{Phagocytosis}\;\mathrm{rate}\;\left(\%\right)=\left(OD_{treated}/OD_{control}\right)\times 100$$

#### 3.4.4. Determination of Nitric Oxide (NO) and Cytokine Levels

RAW 264.7 cells were seeded in 96-well plates (1 × 104 cells/mL, 200 μL/well) and continuously cultured for 12 h. Then, the cells were cultured with different concentrations (100, 50, and 25 μg/mL) of SCSP and LPS (1 μg/mL) for 36 h, the supernatants were collected, and the NO level was determined using the NO assay kit (Microwell plate method, Nanjing Jiancheng Bioengineering Institute, Jiangsu, China). The amounts of secreted TNF-α, IL-6, and IL-1β were determined using a microplate reader and enzyme-linked immunosorbent assay (ELISA) kits (Nanjing Jiancheng Bioengineering Institute, Jiangsu, China) according to the fabricator’s instructions.

#### 3.4.5. Western Blot Analysis

We followed the methods described in Juan Du et al. [53] to measure the proteins levels of iNOS, IκB-α, NF-κB and NLRP3 using western blotting. Total protein of the treated cells was extracted using RIPA lysis buffer, and protein concentration was measured using a bicinchoninic acid (BCA) protein quantification kit (Solarbio, Beijing, China). The proteins were separated using 12% sodium dodecyl sulfate-polyacrylamide gel electrophoresis (SDS-PAGE), transferred to a polyvinylidene fluoride (PVDF) membrane (Millipore, Billerica, MA, USA). Subsequently, the membrane was blocked with 10% non-fat milk for 1.5 h. The PVDF membrane was incubated with diluted specific primary antibodies against iNOS, IκB-α, NF-κB, and NLRP3 at 4 °C with gentle shaking overnight and washed thrice using Tris-buffered saline (TBS) plus Tween-20 (TBST). Finally, the PVDF membrane was incubated with the corresponding secondary antibody (horseradish peroxidase-conjugated goat-anti-rabbit, 1:3000) for 2 h. The intensity of specific bands was detected using enhanced chemiluminescence (ECL) kit and quantified using the Quantity One software (version 4.62). Band intensities were normalized to that of the loading control (β-actin).

### 3.5. Statistical Analysis

All experimental data were expressed as the mean ± standard deviation of at least three independent experiments. Data were analyzed using one-way analysis of variance (ANOVA) of the SPSS software, version 19.0 (SPSS Inc., Chicago, IL, USA). The least significant difference (LSD) was used for post hoc multiple comparisons, and *P* < 0.05 was considered statistically significant for all tests.

## 4. Conclusions

**Figure 9:**
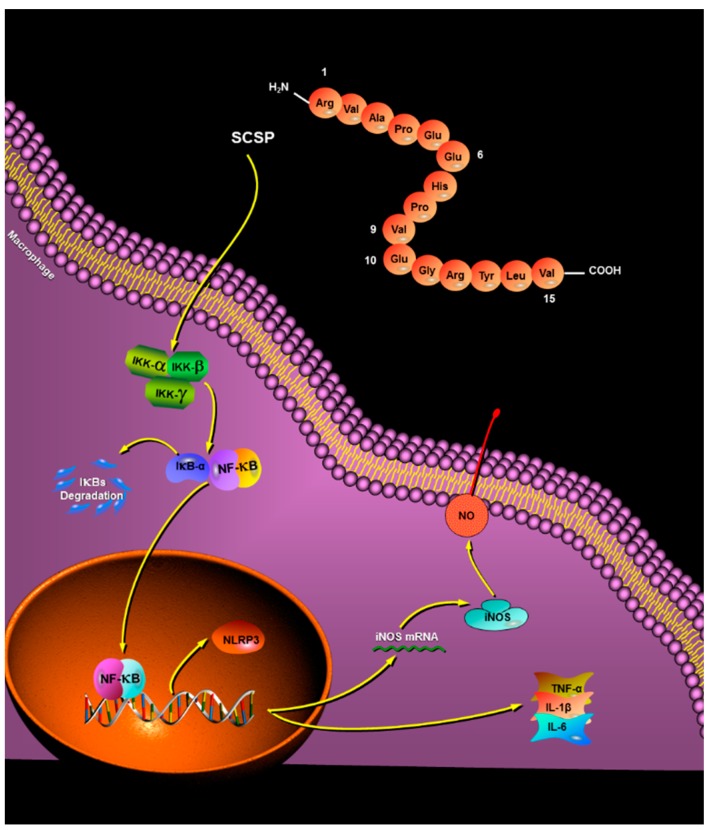
Possible molecular mechanisms of SCSP-induced macrophage activation.

In conclusion, a novel immunomodulatory pentadecapeptide was obtained from the protein hydrolysates of *Cyclina sinensis* using ultrafiltration and chromatographic techniques, and its amino acid sequence was identified to be Arg-Val-Ala-Pro-Glu-Glu-His-Pro-Val-Glu-Gly-Arg-Tyr-Leu-Val (RVAPEEHPVEGRYLV) using automatic Edman degradation. Similar to most peptides with good immunomodulatory activity, the termini of the pentadecapeptide are rich in basic and hydrophobic amino acids, which may explain its immunomodulatory effects. Phagocytosis of RAW264.7 cells was significantly stimulated in a dose-dependent manner. Nitrate reductase assay and western blotting showed that SCSP increased iNOS expression and NO production. Furthermore, TNF-α, IL-1β, and IL-6 levels were elevated. The release of NF-κB from IκB-α was increased, consistent with the protein levels of NLRP3 after SCSP treatment. This suggested that SCSP induced macrophage activation to exert immunomodulatory effects, with the activation of the NF-κB and NLRP3 inflammasome signaling pathways (Figure 9). To validate the current observations, the detailed molecular mechanisms of immunomodulation and their in vivo physiological functions will be investigated in the future. Overall, our results indicated that the immunomodulatory peptide SCSP can be a good candidate for functional foods or dietary supplements.
